# Integrated Analysis of LncRNA-Mediated ceRNA Network in Calcific Aortic Valve Disease

**DOI:** 10.3390/cells11142204

**Published:** 2022-07-14

**Authors:** Long Chen, Ke Wei, Jun Li, Yue Li, Huiqing Cao, Zhe Zheng

**Affiliations:** 1 State Key Laboratory of Cardiovascular Disease, National Center for Cardiovascular Disease, China & Department of Cardiovascular Surgery, Fuwai Hospital, Chinese Academy of Medical Sciences & Peking Union Medical College, Beijing 100006, China; chenlong@fuwai.com (L.C.); weike8596@xinhuamed.com.cn (K.W.); lijun@fuwai.com (J.L.); liyue@fuwai.com (Y.L.); 2 Laboratory of Nucleic Acid Technology, Institute of Molecular Medicine, Peking University, Beijing 100084, China; 3 National Health Commission Key Laboratory of Cardiovascular Regenerative Medicine, Central-China Branch of National Center for Cardiovascular Diseases, Fuwai Central-China Hospital, Beijing 100037, China

**Keywords:** long non-coding RNAs, competitive endogenous RNA networks, bioinformatics, calcific aortic valve disease

## Abstract

Background: The high morbidity and mortality of calcific aortic valve disease (CAVD) represents an unmet clinical need to investigate the molecular mechanisms involved. Evidence suggests that long non-coding RNAs (lncRNAs) can act as competitive endogenous RNAs (ceRNAs) by binding to microRNAs and regulating target genes in cardiovascular diseases. Nevertheless, the role of lncRNAs related ceRNA regulation in CAVD remains unclear. Methods: RNAseq data of human diseased aortic valves were downloaded from GEO data sets (GSE153555, GSE199718), and differentially expressed lncRNAs (DElncRNAs), mRNAs (DEmRNAs) between CAVD and non-calcific aortic valve tissues with limma R package. Gene Ontology (GO) annotation, Kyoto Encyclopedia of Genes and Genomes (KEGG) pathway and Gene Set Enrichment analysis (GSEA) were performed with clusterProfiler and gesaplot2 R package. The pivotal microRNAs were predicted by three databases intersection including TargetScan, MiRwalk, miRDB according to the genes related to the crucial pathways. ENCORI was used to predict targeted lncRNAs of hub microRNAs. We constructed lncRNA-miRNA-mRNA ceRNA network with Cytoscape software. The lncRNAs in ceRNA network were verified by RT-qPCR in human 30 calcific and 20 noncalcified aortic valve tissues. Results: In total, 1739 DEmRNAs and 266 DElncRNAs were identified in CAVD. GO, KEGG pathway, GSEA annotations suggested that most of these genes are enriched in extracellular matrix (ECM)-reporter interaction pathways. The ceRNA networks associated with ECM-reporter interaction are constructed and related lncRNAs including *H19*, *SNHG3* and *ZNF436-AS1* were significant upregulated in human calcific aortic valve tissues, which might be potential therapeutic targets for CAVD. Conclusions: In this study, we proposed a novel lncRNA-miRNA-mRNA ceRNA network related to ECM-reporter interaction pathways, which potentially regulates CAVD progression.

## 1. Introduction

Calcific aortic valve disease (CAVD) is the most common valvulopathy worldwide, the current medical treatment options cannot stop or delay the progression of the life-threatening heart disease [1,2]. CAVD, a progressive disease, involves multiple signaling pathways, lipid accumulation, chronic inflammation, osteogenic differentiation and extracellular matrix (ECM) reorganization pathophysiological process [3]. CAVD is still not sufficiently understood in terms of its biological mechanisms, which limit improvements in therapy. Therefore, further elucidating molecular mechanisms of disease and finding new therapeutic targets to improve the treatment of this life-threatening heart disease.

Non-coding RNAs (ncRNAs), which account for over 90% of the RNA transcripts of the human genome, lack coding protein potential but are considered essential to cell function, including cardiovascular disease [4]. The ncRNAs include microRNAs, 21–24 base pairs, and long noncoding RNAs (lncRNAs), longer than 200 base pairs [5]. The lncRNAs are important to epigenetic, transcriptional and posttranscriptional regulation of gene expression, which are an essential participant in the progression of CAVD [6,7,8]. The competitive endogenous RNA (ceRNA) hypothesis was first proposed by Salmena et al. [9]. As ceRNAs, by combining common miRNAs some lncRNAs can influence downstream mRNA expression. There is increasing evidence that lncRNAs can act as ceRNAs by competing for shared miRNAs to regulate target mRNAs expression [10]. In Supplementary Figure S1, a schematic diagram shows the lncRNA-mediated regulatory network of ceRNA. A number of dysregulated lncRNAs exert a key role in the progression of CAVD [6,11,12], which demonstrate their potential roles in osteogenic differentiation of valvular cells and ECM remodeling. A growing body of research suggests that lncRNAs could be involved in the development of various cardiovascular diseases, such as atherosclerosis [11], coronary artery disease [12], and heart failure [13]. However, to date, research on the role of lncRNA-related ceRNA regulatory networks in CAVD is rare.

In this study, a genome-wide expression profile of lncRNAs and mRNAs of patients with CAVD was analyzed through a public functional genomics data repository Gene Expression Omnibus (GEO). Moreover, comprehensive analysis was used to create a CAVD-specific ceRNA network. The ceRNA network will help discovery of new therapeutic targets and pathways for treating patients with CAVD. We verified these expression of theses lncRNAs and miRNAs in human calcific aortic valve tissues and normal control. Further studies are required to validate the molecular mechanism of these lncRNAs ceRNA network as therapeutic targets for CAVD.

## 2. Materials and Methods

### 2.1. Data Collection and Process

The RNAseq data, lncRNA seq data and corresponding CAVD clinical data are downloaded from GEO database. A total of 20 CAVD samples and 22 noncalcified aortic valve tissues samples are collected. The original expression matrix is normalized and identified out differentially expressed genes with limma R package [14]. GSE153555 contains 10 calcified and 10 noncalcified valve tissues samples for differentially expressed mRNA (DEmRNAs) analysis. GSE199718 contains 10 calcific and 12 noncalcified aortic valve tissues for differentially expressed lncRNAs (DElncRNAs). The differentially expressed genes are identified based on the following selection criteria: an adjusted *p*-value < 0.05 and log2 (fold change) greater than 1 between calcific and noncalcified aortic valve samples.

### 2.2. Enrichment Analysis

Gene set enrichment analysis (GSEA) sequences the genes based on the differential expression degree of the two samples and then determines whether the preset gene set is enriched at the top or bottom of the sequencing table, which reveals several shared biological pathways [15]. The GSEA tests the expression of genomewide expression profiles instead of individual genes, and can capture more sensitive changes in expression using annotations from hallmark gene sets to reduce noise and redundancy [16]. ClusterProfiler package in R software (v3.6.2, Ross Ihaka and Robert Gentleman, Auckland, New Zealand) is conducted to investigate the DEmRNAs in CAVD, including molecular function (MF), biological process (BF), cell composition (CC) and Kyoto Encyclopedia of Genes and Genomes (KEGG) pathway [17].

### 2.3. Protein-Protein Interaction (PPI) Network Analysis and Gene Cluster Identification

The protein network interaction diagram is created using DEmRNAs from CAVD samples uploaded to STRING [18]. The result of STRING analysis is imported into Cytocape (v.3.8.0, Paul Shannon and colleagues, Seattle, WA, USA) [19], and cluster analysis of differential genes is performed with Molecular Complex Detection (MCODE) plug-in [20] and Gene Ontology (GO) terms and KEGG pathways are analyzed with ClueGO plug-in to create a functionally organized GO/pathway term network [21]. STRING is conducted to draw PPI network and the biological processes that the genes in the gene cluster with the highest scores are involved in. The screened gene cluster is analyzed with Network Analyst (v3.0, Guangyan Zhou and colleagues, Montreal, QC, Canada) to further verify the biological processes and pathways [22].

### 2.4. Prediction of Hub miRNAs and Investigation of mRNA-miRNA Interaction Network Analysis

Genes associated with pivotal pathway are selected and conducted with miRWalk 3.0 database to predict its targeted miRNAs [23]. To verify the accuracy of the predicted miRNAs, TargetScan [24], miRWalk and MiRDB [25] databases are used to do intersection. Subsequently, we integrate the intersection of targeted miRNAs with DEmRNAs, which are further processed with Cytoscape.

### 2.5. MiRNAs-lncRNAs Interaction Network Analysis

The Encyclopedia of RNA Interactomes (ENCORI) website is utilized to predict the target molecules lncRNAs of the selected miRNAs [26], which were taken the intersection with DElncRNAs from GSE199718. The obtained data were constructed to a network further with Cytoscape.

### 2.6. Real-Time Polymerase Chain Reaction

Three candidate lncRNAs, which may be the therapeutic targets for CAVD, were screened for further confirmation. Twenty control noncalcified aortic valve tissues from patients undergoing heart translation for various cardiomyopathy and thirty calcified aortic valve tissues from patients undergoing aortic valve replacement for CAVD were collected for further study (clinical characteristics of patients are shown in Supplementary Table S1). The study was performed with approval of the institutional Ethics Committees of the Fuwai hospital, Chinese Academy of Medical Sciences and complied with the Declaration of Helsinki (No.2012-404). Before participating in the study, the written informed consent was obtained from all patients.

The RNA was extracted from aortic valve tissues using Trizol reagent (Invitrogen Corporation, Carlsbad, CA, USA), and then was reverse transcribed into cDNA with PrimeScript RT Master Mix (RR036A, Takara Bio Inc., Beijing, China) at 37 °C for 15 min and 85 °C for 5 s. Next, the real-time quantitative PCR was performed with TB Green Premix Ex TB Green Premix Ex Taq II (RR820A, Takara Bio Inc., Beijing, China) based on the QuantStudio™ 5 System (Applied Biosystems Inc., Foster, CA, USA). The relative expression of each selected lncRNAs between two groups was determined by the 2^−ΔΔCt^ method. The primer sequences for lncRNAs utilized in the study are listed in Supplementary Table S2. The primer sequences for miRNAs were purchased from RiboBio (Guangzhou, China).

### 2.7. Statistical Analysis

Continuous data are presented as mean value ± standard error of the mean (SEM). The normality of the distribution of continuous data was confirmed by Shapiro–Wilk test and was visualized by a Q–Q plot. The Levene test was used to confirm the homogeneity of variance of continuous data. For normally distributed data, comparisons between the two groups were evaluated for significance using the unpaired Student’s t-test. Statistical significance was set at *p* < 0.05.

## 3. Results

### 3.1. Processing of Sample Data and Identification of Differentially Expressed Genes

**Figure 1:**
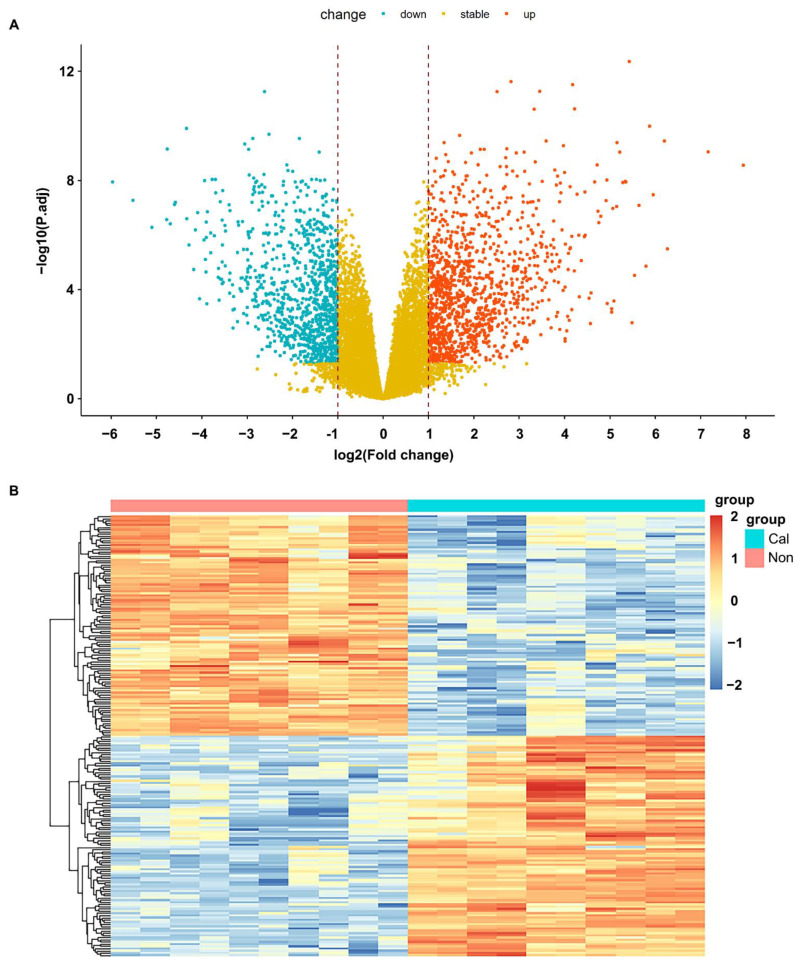
Differentially expressed genes in calcified and noncalcified aortic valve tissue. (**A**) The volcano plot and (**B**) hierarchical cluster heat map of differentially expressed genes.

Based on the expression matrix and sample information of GSE153453, 1831 differentially expressed genes from CAVD patients, including 1114 upregulated genes and 717 downregulated genes. The following were the screening criteria for differentially expressed genes: log2 fold change greater than one and adjusted *p* value less than 0.05. On the basis of variance of gene expression in the CAVD, the volcano plot was showed in Figure 1A and the hierarchical cluster analysis and heat mapping was presented in Figure 1B.

### 3.2. Functional Enrichment Analysis

**Figure 2:**
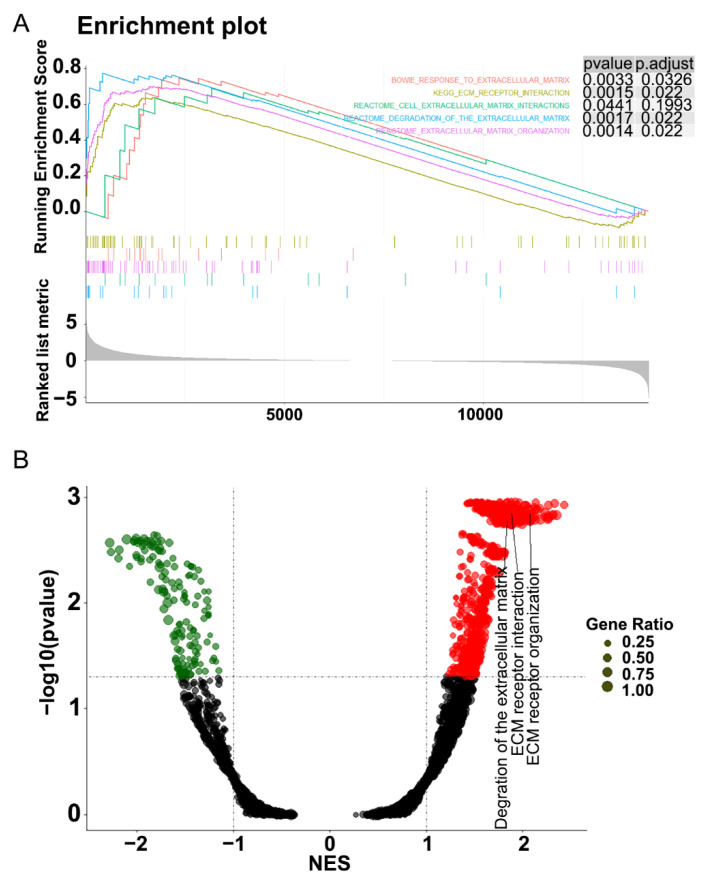
Gene set enrichment analysis (GSEA) of the whole gene expression of the calcified and noncalcified aortic valve tissues. (**A**) Extracellular matrix (ECM) receptor interaction, cell ECM interactions, degradation of the ECM and ECM organization were significantly enriched. (**B**) The volcano plot of GSEA revealed degradation of ECM, ECM receptor interaction and ECM organization were significantly enriched.

To explore the functions of gene expression, gene set enrichment analysis (GSEA), gene ontology (GO) and Kyoto Encyclopedia of Genes and Genomes (KEGG), pathway enrichment analyses were used to analyze the samples’ genes expression. Firstly, all genes expression in calcified and noncalcified aortic valve tissues was analyzed with GSEA Base R package, and genes were investigated at the overall level of expression profile using the hallmark gene set database. The significantly enriched gene sets are defined as adjusted *p* value < 0.05. As illustrated in Figure 2, gene sets were significantly enriched in extracellular matrix (ECM)-receptor interaction and ECM-related functions in CAVD samples.

**Figure 3:**
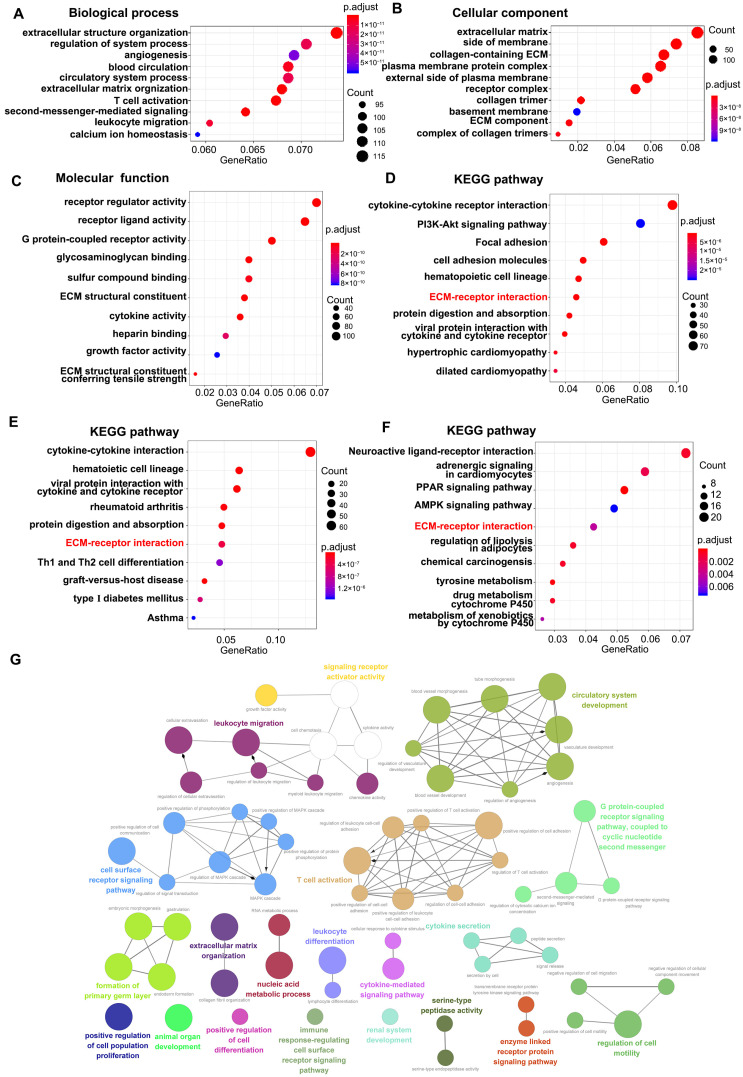
Gene ontology and Kyoto Encyclopedia of Genes and Genomes (KEGG) enrichment of differentially expressed genes. (**A**) Biological process revealed extracellular structure organization and extracellular matrix (ECM) organization were enriched. (**B**) ECM-related cellular components were significant enriched. (**C**) Among molecular function, the receptor regulator activity, receptor ligand activity, ECM structural constituent, and ECM structural conferring tensile strength were enriched. KEGG enrichment analysis revealed the ECM-receptor interaction pathway was significant enriched in all 1831 DEmRNAs (**D**), 1114 upregulated DEmRNAs (**E**) and 717 downregulated DEmRNAs (**F**). (**G**) The ClueGO plug-in was used to investigate the interaction networks of enriched biological processes.

The ClusterProfiler R package was used to perform GO and KEGG pathway enrichment analysis on 1831 DEmRNAs from CAVD samples. GO enrichment analysis indicated that the differentially expressed genes were mainly associated with extracellular structure organization and extracellular matrix organization in the biological process (Figure 3A). ECM, collagen-containing ECM and ECM-related cellular components were significantly enriched (Figure 3B). Among top 10 molecular function, the receptor regulator activity, receptor ligand activity, G protein-coupled receptor activity, ECM structural constituent, and ECM structural conferring tensile strength were significantly enriched (Figure 3C). KEGG enrichment analysis revealed the ECM-receptor interaction pathway was significant enriched in all 1831 DEmRNAs (Figure 3D), 1114 upregulated DEmRNAs (Figure 3E) and 717 downregulated DEmRNAs (Figure 3F). In addition, the CLUEGO plug-in of Cytoscape was used to show the interaction network of biological process. The results also presented that cell surface receptor signaling pathway, signaling receptor activator activity, and ECM organization et.al were significant enriched (Figure 3G). These data revealed that the differentially expressed genes in CAVD were linked to ECM-receptor interaction pathway and ECM-remodeling.

### 3.3. Construction of Protein-Protein Interaction (PPI) Network and Investigation of Gene Clusters Participating in ECM-Related Biological Pathway

**Figure 4:**
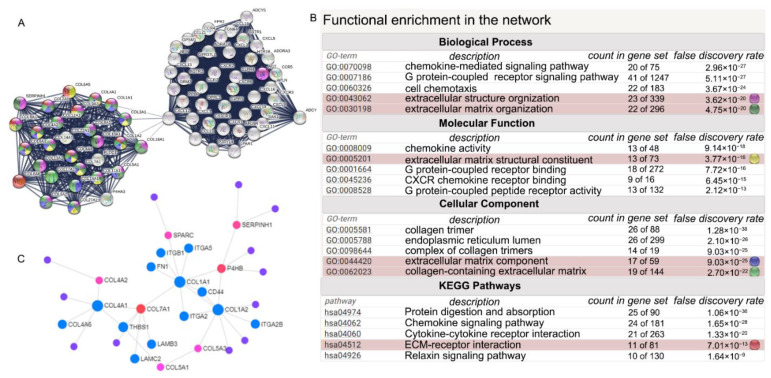
Functional enrichment of the gene cluster1 with the highest score of MCODE. (**A**) STRING reveals the interaction of genes in cluster 1. (**B**) Functional enrichment analyses of genes in cluster 1. (**C**) Network Analyst is used to validate the enrichment results and genes involved in extracellular matrix (ECM)-receptor interaction are noted in blue. The red nodes indicate protein digestion and absorption pathway and the violet nodes represent the genes involved in cytokine-cytokine receptor interaction.

To filter out the hub genes from DEmRNAs in CAVD samples, 1831 DEmRNAs were uploaded to STRING website for further analysis, and the PPI network with 1669 nodes and 16,190 edges were achieved. Next, the PPI network was uploaded in Cytoscape and MCODE plug-in was used to analyze the network to identify gene clusters with top 5 scores (Supplementary Table S3). The 72 genes in the cluster 1 with the highest score were chosen for GO and KEGG enrichment analysis, which demonstrated that the genes in this cluster were mainly involved in ECM-receptor interaction pathway and ECM-associated function. STRING and Network Analyst database were utilized to investigate the hub genes in gene cluster 1, and findings showed that gene cluster 1 mainly was involved in cytokine-cytokine receptor interaction, ECM-receptor interaction pathway, extracellular structure organization, ECM organization biological process, ECM structural constituent, ECM component molecular function, collagen-containing ECM cellular component all of which had statistical significance based on FDR values, and then the linked genes were constructed into network in STRING (Figure 4A,B). To further identify how gene cluster 1 also plays a significant role in the CAVD, we also uploaded gene cluster 1 into Network Analyst and found the molecules involved in ECM-receptor interaction pathway were highlighted as shown in Figure 4C.

### 3.4. Further miRNA Mining and mRNA-miRNA Interaction Network Analysis

**Figure 5:**
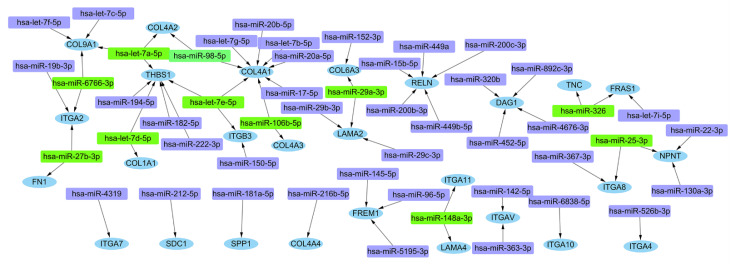
Interaction network between genes involved in extracellular matrix and its targeted miRNAs. Genes are colored in blue; miRNAs are colored in violet; and miRNAs targeting over two genes simultaneously are colored in green.

Among the seventy-two hub genes of gene cluster 1, thirty-seven differential expressed genes related to ECM-receptor interaction pathway were screened out. Then, miRWalk 3.0 program was used for mRNA-miRNA interaction analysis. The prediction results were chosen from the intersection of miRNAs predicted by the TargetScan, miRDB, and miRWalk databases. The selection criteria were set as *p* < 0.05, target gene binding region was 3′UTR and seed sequence length was at least 7 mer. The mRNA-miRNAs interaction network was constructed with Cytoscape (Figure 5). The miRNAs with large number of shared DEmRNAs (≥2) were chosen as hub miRNAs (Supplementary Table S4).

### 3.5. Construction of lncRNA-miRNA-mRNA ceRNA Network in CAVD

**Figure 6:**
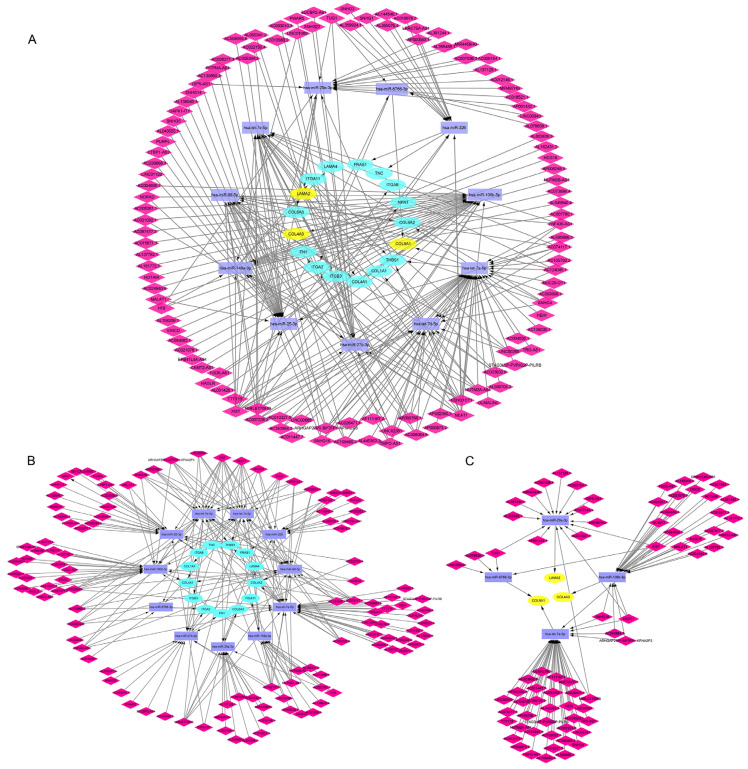
LncRNA-miRNA-mRNA network in CAVD. (**A**) The differential mRNAs involved in extracellular matrix-receptor interaction were constructed the complementary endogenous networks. The up-regulated mRNAs (**B**) and down-regulated mRNAs (**C**) separately were constructed the complementary endogenous networks. The blue nodes indicate the up-regulated mRNAs involved in extracellular matrix-receptor interaction pathway, the yellow nodes indicate the down-regulated mRNAs, the violet nodes represent the hub miRNAs and the magenta nodes indicate the lncRNAs.

The corresponding lncRNAs of hub miRNAs were predicted with ENCORI software [26]. The selection conditions were set the highest reliability (very high stringency of CLIP data ≥ 5) and 113 lncRNAs were predicted to interact with the hub miRNAs. The number of CLIP sites and target sequence in each of the lncRNA-miRNA relations within the ENCORI databases are shown in Supplementary Table S5. The lncRNA-miRNA-mRNA ceRNA network was constructed with Cytoscape as shown in Figure 6A. In order to obtained more detailed biological insights, we separately to analyze that mRNAs were up or down-regulated networks in CAVD (Figure 6B,C).

### 3.6. Verification of the Potential lncRNAs Expression in ceRNA Network

**Figure 7:**
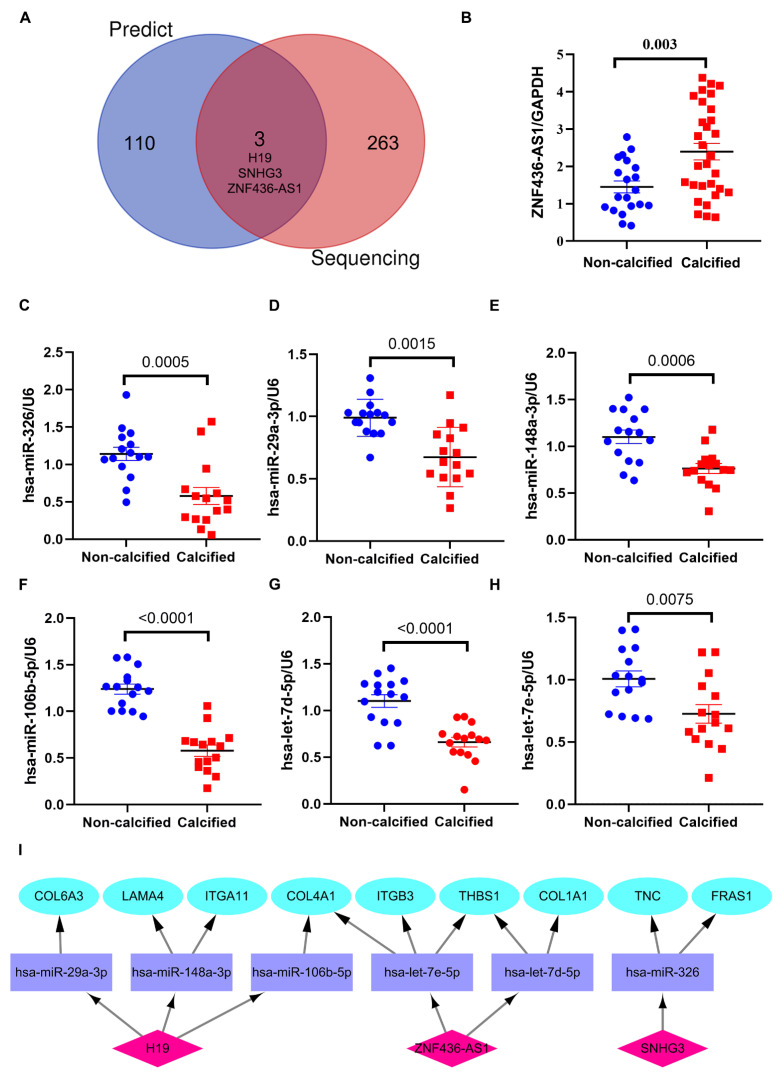
Verification of the lncRNAs and microRNAs in the ceRNA network. (**A**) The Venn diagram indicates three lncRNAs (*H19, SNHG3 and ZNF436-AS1*) in competitive endogenous networks were verified by another sequencing data. (**B**) The lncRNA *ZNF436-AS1* was highly expressed in calcified aortic valves (*n* = 30) than that in control (*n* = 20). (**C**–**H**) RT-qPCR results show that the expression of has-miR-326, has-miR-29a-3p, has-miR-148-3p, has-miR-106b-5p, has-let-7d-5p and has-let-7e-5p in human calcified aortic valves (*n* = 15) were obviously lower than that non-calcified control (*n* = 15). (**I**) The competitive endogenous network including validated up-regulated lncRNAs (H19, SNHG3 and ZNF436-AS1), down-regulated miRNAs (has-miR-326, has-miR-29a-3p, has-miR-148a-3p, has-miR-106b-5p, has-let-7d-5p, has-let-7e-5p) and up-regulated mRNAs (*COL4A1, COL1A1, COL6A3, THBS1, ITGB3, LAMA4, ITGA11, FRAS1, TNC*) in the calcified aortic valves were constructed. The magenta nodes, violet nodes and blued nodes indicate up-regulated lncRNAs, down-regulated miRNAs and up-regulated mRNAs, separately. Unpaired 2-tailed Student’s *t*-test.

To verify the potential 113 lncRNAs in the ceRNA network, we first analyzed the DElncRNAs in the GSE199718 and screened out that 152 lncRNAs were upregulated and 114 lncRNAs were significantly downregulated in CAVD with the criteria: Log2 fold change > 0.6 and adjusted *p* value < 0.05. We found 3 DElncRNAs in the ceRNA network: *H19*, *SNHG3* and *ZNF436-AS1* (Figure 7A). H19 was reported that DNA hypomethylation in the promoter region of *H19* during CAVD leads to an overexpression of this lncRNA, resulting in promoting osteogenic transition of VICs [6]. Then, we also verified these 3 lncRNAs in 50 human samples (30 cases of calcified and 20 noncalcified aortic valve tissues) using RT-qPCR and the expression of *H19* (*p* = 0.0001), *SNHG3* (*p* < 0.0001) and *ZNF436-AS1* (*p* = 0.003) (Figure 7B) in calcified aortic valve tissues were significantly higher than that of non-calcified control. Our previous research found that SNHG3 was increased in calcified aortic valves, which prompted osteogenic differentiation of VICs via upregulation of the BMP2 pathway [27]. Additionally, we also detected the expression of predicted miRNAs regulated by validated lncRNAs *(H19*, *SNHG3* and *ZNF436-AS1*) in human aortic valves using RT-qPCR. We also found that has-miR-326 (*p* = 0.0005), has-miR-29a-3p (*p* = 0.0002), has-miR-148a-3p (*p* = 0.0006), has-miR-106b-5p (*p*<0.0001), has-let-7d-5p (*p* < 0.0001), and has-let-7e-5p (*p* = 0.0075) were significantly down-regulated in calcified aortic valve tissues (Figure 6C–H). Thus, we constructed a competitive endogenous network including validated up-regulated lncRNAs (*H19*, *SNHG3* and *ZNF436-AS1*), down-regulated miRNAs (has-miR-326, has-miR-29a-3p, has-miR-148a-3p, has-miR-106b-5p, has-let-7d-5p, has-let-7e-5p) and up-regulated mRNAs (*COL4A1*, *COL1A1*, *COL6A3*, *THBS1*, *ITGB3*, *LAMA4*, *ITGA11*, *FRAS1*, *TNC*) in the calcified aortic valves (Figure 6I).

## 4. Discussion

Calcific aortic valve disease is the most frequent valvular heart disease among developed countries and the elderly [2]. Given the severe morbidity and mortality associated with CAVD, identifying specific biomarkers and therapeutic targets for the diagnosis and treatment of patients with CAVD is an urgent concern. In this study, we used CAVD expression data downloaded from GEO databases and screened out 1831 differential expressed genes including 1114 upregulated genes and 717 downregulated genes. Then, GSEA, GO and KEGG enrichment analysis showed these genes are mainly participating in ECM component and ECM-receptor interaction pathway. Our study focused on the DEmRNAs related to ECM in CAVD. Based on the DEmRNAs associated with ECM, we constructed a miRNA-mRNA network with 77 nodes and 63 edges. The miRNAs that can target more than 2 DEmRNAs related to ECM act as the hub miRNAs, which were utilized to predict the lncRNAs with ENCORI software. We constructed a CAVD-specific ceRNAs network with these hub DEmRNAs, miRNAs and lncRNAs. Importantly, three lncRNAs were validated in a completely independent lncRNA sequencing data from GEO database (GSE199718) and we also verified that lncRNAs (*H19*, *SNHG3* and *ZNF436-AS1*) were significantly upregulated and miRNAs (has-miR-326, has-miR-29a-3p, has-miR-148a-3p, has-miR-106b-5p, has-let-7d-5p, has-let-7e-5p) were significantly downregulated in human calcified aortic valves tissues.

CAVD is characterized by changes in the organization, composition and mechanical properties of the ECM [28]. According to growing evidence, ECM maladaptations not only result in valve cell dysfunction, but they also contribute to the development of CAVD [29]. The function and pathway enrichment showed ECM receptor interaction signaling pathways were significantly enriched in our analysis. Compared to other microarray datasets, enrichment analysis found PI3K/AKT signaling pathway in an AMPK-dependent manner was inhibited in CAVD [30], our enrichment results also revealed that the downregulated DEmRNAs could enriched in AMPK signaling pathway. Among the DEmRNAs involved in the lncRNA-miRNA-mRNA ceRNA network, COL4A1, COL4A2, COL4A3, COL1A1, COL6A3 and COL9A1 were belong to constitute of type I, IV, VI and IX collagen, which are structural proteins in the ECM. The aortic valve is composed of three layers: the fibrosa, spongiosa and ventricularis. These collagens are present in all three layers. Type I collagen, localized in the myofibroblasts adjacent to calcified aortic valve nodules, is significantly increased [31]. COL6A3 is the main component of type VI collagen, which is involved in the progression of calcific aortic valve stenosis [32]. The localization of collagen VI around the calcified nodules is critical for the spreading of the mineralization of aortic valve, which brings about calcium and hydroxyproline accumulations on human osteoblast-like cells and promotes osteogenic differentiation of VICs during CAVD progression [33]. COL9A1, encoding one of three alpha chains of type IX collagen, is a minor collagen component of hyaline cartilage. Type IX collagen is attached to the surface of type II collagen fibrils covalently in the ECM special for cartilage [34]. The expression of type IX collagen mRNA in the mouse heart has also been detected throughout embryonic development [35]. The function of type IX collagen in aortic valve calcification remains unknown and was not studied further in the present. Dysregulation of these collagen components can alter ECM elasticity in valve tissue, which can regulate the myofibroblast differentiation of valvular interstitial cells (VICs) [36]. Additionally, the effect of ECM elasticity owing to collagens disorganized contributes to VICs calcification and osteogenic differentiation.

TNC, a hexametric ECM glycoprotein, is upregulated significantly and moved from the basement membrane to the interstitial space around the calcified aortic valve area [37,38]. TGF-β, BMPs and TNF-α can induce TNC expression in the VICs of diseased valves [39,40,41]. TNC can promote ALP activity, collagen synthesis around calcifying regions and ECM remodeling. TNC can also modulate VICs adhesion, phenotypic expression and calcification through syndecan-4 and its downstream focal adhesion kinase and RhoA signaling [42,43]. ITGA2, ITGA8 and ITGB3 belong to integrin, which are the transmembrane receptor for collagens and related proteins. Integrin alpha-2 regulates platelets and other cells adhesion to collagens, collagen and collagenase gene expression, force generation and organization of newly synthesized ECM [44]. ITGA8 and ITGB3 can recognize the sequence R-G-D in a wide array of ligands including TNC, FN1, SPP1, TGFB1, NPNT and VTN. Specific integrins interact between VICs and ECM modulating calcification [45]. ITGB3, encoding integrin alpha-V/beta-3, interacts with collagen I and fibronectin to increase motility, invasiveness, and remodeling, as it does in atrioventricular cushion mesenchymal cells via Rho/PI3-kinase signal [46].

*H19* was the most significant upregulated DElncRNAs in the ceRNA network in CAVD. Mathieu et al. revealed that a dysregulation of DNA methylation in the promoter of H19, which promotes an osteogenic differentiation of VICs by interfering with expression of NOTCH1 during CAVD [6]. *HOTAIR* was reported to be downregulated in BAVs and VICs exposed to cyclic stretch. *HOTAIR* is involved in aortic valve calcification and repressed by WNT β-CATENIN signaling [47]. TUG1 has been shown to sponge miR-204-5p to promote osteogenic differentiation of VICs via upregulating RUNX2 in CAVD [7]. LncRNA *MALAT1* was observed to sponge miR-204 to promote osteogenic differentiation of VICs via upregulating Smad4 in CAVD [8]. LncRNA *OIP5-AS1* was downregulated and crosstalk with miR-137 and TWIST11 in CAVD [48]. Some studies have reported that SNHG3 can mediate molecular markers related to CAVD, such as *NOTCH1*, *RUNX2* in some tumors [49,50]. Relevant studies on function of other lncRNAs in the ceRNA networks do not exist in the literature.

Based on our current study, we constructed an ECM related ceRNA regulatory network and verified the expression of lncRNAs including *H19*, SNHG3 and *ZNF436-AS1* in human aortic valve tissues, which have strong potential for use as novel biomarkers and therapeutic targets. In recent years, some studies have shown that lncRNAs can be used as a biomarker for diagnosing diseases. Yang Y et.al reported that lncRNA CoreMarker could be used as biomarker for diagnosis of coronary artery disease [51]. LncRNA-NRF was reported to be a potential biomarker of heart failure after acute myocardial infarction [52]. Mathieu and colleagues reported that a dysregulation of DNA methylation in the promoter of *H19* during CAVD upregulates its expression, which promotes an osteogenic program by interfering with the expression of NOTCH1 [6]. Our previous research also found that *SNHG3* is upregulated in the calcific aortic valves and acts as a decoy lncRNA, interacting with EZH2 to suppress the H3K27 tri-methylation of the BMP2 promoter, resulting in the upregulation of the BMP2 signaling pathway, thereby promoting osteogenic differentiation of valve interstitial cells in CAVD progression [27]. Thus, *H19* and *SNHG3* could represent a novel target in CAVD to decrease the osteogenic activity in the aortic valve. There is currently no research on the role and mechanism of *ZNF436-AS1* in CAVD. In addition, we also verified a series of miRNAs sponged by these lncRNA. Hence, our results establish the groundwork for further research to validate the underlying biological regulatory mechanism.

Several limitations remain in this study. First, for the validation analysis, control samples from transplant recipient hearts, which may not represent “healthy” valves, and the diseased valves from the patients undergoing aortic valve replacement surgery due to severe calcification. Additionally, the results from public available databases and bioinformatic analysis require further biological proof-of-concept studies to verify. Under these conditions, further cellular and animal experiments are necessary in the future to confirm these molecular mechanisms of ceRNAs in CAVD progression.

## 5. Conclusions

In this study, we proposed a novel lncRNA-miRNA-mRNA ceRNA network related to ECM-receptor interaction pathways and ECM components, which potentially regulates CAVD progression.

## Data Availability

The datasets presented in this study can be found in online repositories Gene Expression Omnibus database.

## Supplementary Materials

The following supporting information can be downloaded at: https://www.mdpi.com/article/10.3390/cells11142204/s1, Figure S1: The schematic diagram of lncRNA-mediated ceRNA regulatory network; Table S1: Clinical characteristics of patients for RT-qPCR analysis; Table S2: The primer sequences for real-time polymerase chain reaction; Table S3: The differentiational expressed mRNAs downloaded from STRING was processed using MCODE for further gene cluster analysis. An analysis of gene clusters with top 5 scores was conducted and presented in a table; Table S4: The hub miRNAs and Its target genes. Table S5: The number of CLIP sites and target sequence in each of the lncRNA-miRNA relations within the ENCORI databases.

## References

[1] Yadgir S., Johnson C.O., Aboyans V., Adebayo O.M., Adedoyin R.A., Afarideh M., Alahdab F., Alashi A., Alipour V., Arabloo J. (2020). Global, Regional, and National Burden of Calcific Aortic Valve and Degenerative Mitral Valve Diseases, 1990–2017. Circulation.

[2] Thaden J.J., Nkomo V.T., Enriquez-Sarano M. (2014). The global burden of aortic stenosis. Prog. Cardiovasc. Dis..

[3] Goody P.R., Hosen M.R., Christmann D., Niepmann S.T., Zietzer A., Adam M., Bonner F., Zimmer S., Nickenig G., Jansen F. (2020). Aortic Valve Stenosis: From Basic Mechanisms to Novel Therapeutic Targets. Arter. Thromb. Vasc. Biol..

[4] Uchida S., Dimmeler S. (2015). Long noncoding RNAs in cardiovascular diseases. Circ. Res..

[5] Barwari T., Joshi A., Mayr M. (2016). MicroRNAs in Cardiovascular Disease. J. Am. Coll. Cardiol..

[6] Hadji F., Boulanger M.C., Guay S.P., Gaudreault N., Amellah S., Mkannez G., Bouchareb R., Marchand J.T., Nsaibia M.J., Guauque-Olarte S. (2016). Altered DNA Methylation of Long Noncoding RNA H19 in Calcific Aortic Valve Disease Promotes Mineralization by Silencing NOTCH1. Circulation.

[7] Yu C., Li L., Xie F., Guo S., Liu F., Dong N., Wang Y. (2018). LncRNA TUG1 sponges miR-204-5p to promote osteoblast differentiation through upregulating Runx2 in aortic valve calcification. Cardiovasc. Res..

[8] Xiao X., Zhou T., Guo S., Guo C., Zhang Q., Dong N., Wang Y. (2017). LncRNA MALAT1 sponges miR-204 to promote osteoblast differentiation of human aortic valve interstitial cells through up-regulating Smad4. Int. J. Cardiol..

[9] Salmena L., Poliseno L., Tay Y., Kats L., Pandolfi P.P. (2011). A ceRNA hypothesis: The Rosetta Stone of a hidden RNA language?. Cell.

[10] He L., Chen Y., Hao S., Qian J. (2018). Uncovering novel landscape of cardiovascular diseases and therapeutic targets for cardioprotection via long noncoding RNA-miRNA-mRNA axes. Epigenomics.

[11] Yu X.H., Deng W.Y., Chen J.J., Xu X.D., Liu X.X., Chen L., Shi M.W., Liu Q.X., Tao M., Ren K. (2020). LncRNA kcnq1ot1 promotes lipid accumulation and accelerates atherosclerosis via functioning as a ceRNA through the miR-452-3p/HDAC3/ABCA1 axis. Cell Death Dis..

[12] Bian W., Jiang X.X., Wang Z., Zhu Y.R., Zhang H., Li X., Liu Z., Xiong J., Zhang D.M. (2021). Comprehensive analysis of the ceRNA network in coronary artery disease. Sci. Rep..

[13] Song C., Zhang J., Liu Y., Hu Y., Feng C., Shi P., Zhang Y., Wang L., Xie Y., Zhang M. (2022). Characterization and Validation of ceRNA-Mediated Pathway-Pathway Crosstalk Networks Across Eight Major Cardiovascular Diseases. Front. Cell Dev. Biol..

[14] Ritchie M.E., Phipson B., Wu D., Hu Y., Law C.W., Shi W., Smyth G.K. (2015). Limma powers differential expression analyses for RNA-sequencing and microarray studies. Nucleic Acids. Res..

[15] Subramanian A., Tamayo P., Mootha V.K., Mukherjee S., Ebert B.L., Gillette M.A., Paulovich A., Pomeroy S.L., Golub T.R., Lander E.S. (2005). Gene set enrichment analysis: A knowledge-based approach for interpreting genome-wide expression profiles. Proc. Natl. Acad. Sci. USA.

[16] Liberzon A., Birger C., Thorvaldsdottir H., Ghandi M., Mesirov J.P., Tamayo P. (2015). The Molecular Signatures Database (MSigDB) hallmark gene set collection. Cell Syst..

[17] Yu G., Wang L.G., Han Y., He Q.Y. (2012). ClusterProfiler: An R package for comparing biological themes among gene clusters. Omics J. Integr. Biol..

[18] Szklarczyk D., Morris J.H., Cook H., Kuhn M., Wyder S., Simonovic M., Santos A., Doncheva N.T., Roth A., Bork P. (2017). The STRING database in 2017: Quality-controlled protein-protein association networks, made broadly accessible. Nucleic Acids Res..

[19] Shannon P., Markiel A., Ozier O., Baliga N.S., Wang J.T., Ramage D., Amin N., Schwikowski B., Ideker T. (2003). Cytoscape: A software environment for integrated models of biomolecular interaction networks. Genome Res..

[20] Bader G.D., Hogue C.W. (2003). An automated method for finding molecular complexes in large protein interaction networks. BMC Bioinform..

[21] Bindea G., Mlecnik B., Hackl H., Charoentong P., Tosolini M., Kirilovsky A., Fridman W.H., Pages F., Trajanoski Z., Galon J. (2009). ClueGO: A Cytoscape plug-in to decipher functionally grouped gene ontology and pathway annotation networks. Bioinformatics.

[22] Zhou G., Soufan O., Ewald J., Hancock R.E.W., Basu N., Xia J. (2019). NetworkAnalyst 3.0: A visual analytics platform for comprehensive gene expression profiling and meta-analysis. Nucleic Acids Res..

[23] Sticht C., De La Torre C., Parveen A., Gretz N. (2018). MiRWalk: An online resource for prediction of microRNA binding sites. PLoS ONE.

[24] Agarwal V., Bell G.W., Nam J.W., Bartel D.P. (2015). Predicting effective microRNA target sites in mammalian mRNAs. eLife.

[25] Chen Y., Wang X. (2020). miRDB: An online database for prediction of functional microRNA targets. Nucleic Acids Res..

[26] Li J.H., Liu S., Zhou H., Qu L.H., Yang J.H. (2014). starBase v2.0: Decoding miRNA-ceRNA, miRNA-ncRNA and protein-RNA interaction networks from large-scale CLIP-Seq data. Nucleic Acids Res..

[27] Long C., Hanning L., Cheng S., Jianqiu P., Jun L., Yue L., Ke W., Xiaoyi W., Peng W., Fangzhou L. A Novel Long Noncoding RNA SNHG3 Promotes Osteoblast Differentiation through BMP2 Upregulation in Aortic Valve Calcification. JACC Basic Transl. Sci..

[28] Chen J.H., Simmons C.A. (2011). Cell-matrix interactions in the pathobiology of calcific aortic valve disease: Critical roles for matricellular, matricrine, and matrix mechanics cues. Circ. Res..

[29] Di Vito A., Donato A., Presta I., Mancuso T., Brunetti F.S., Mastroroberto P., Amorosi A., Malara N., Donato G. (2021). Extracellular Matrix in Calcific Aortic Valve Disease: Architecture, Dynamic and Perspectives. Int. J. Mol. Sci..

[30] En Q., Zeping H., Yuetang W., Xu W., Wei W. (2021). Metformin alleviates the calcification of aortic valve interstitial cells through activating the PI3K/AKT pathway in an AMPK dependent way. Mol. Med..

[31] Eriksen H.A., Satta J., Risteli J., Veijola M., Vare P., Soini Y. (2006). Type I and type III collagen synthesis and composition in the valve matrix in aortic valve stenosis. Atherosclerosis.

[32] White J.F., Werkmeister J.A., Hilbert S.L., Ramshaw J.A. (2010). Heart valve collagens: Cross-species comparison using immunohistological methods. J. Heart Valve Dis..

[33] Mourino-Alvarez L., Iloro I., de la Cuesta F., Azkargorta M., Sastre-Oliva T., Escobes I., Lopez-Almodovar L.F., Sanchez P.L., Urreta H., Fernandez-Aviles F. (2016). MALDI-Imaging Mass Spectrometry: A step forward in the anatomopathological characterization of stenotic aortic valve tissue. Sci. Rep..

[34] Durand A.L., Dufour A., Aubert-Foucher E., Oger-Desfeux C., Pasdeloup M., Lustig S., Servien E., Vaz G., Perrier-Groult E., Mallein-Gerin F. (2020). The Lysine Specific Demethylase-1 Negatively Regulates the COL9A1 Gene in Human Articular Chondrocytes. Int. J. Mol. Sci..

[35] Liu C.Y., Olsen B.R., Kao W.W. (1993). Developmental patterns of two α 1(IX) collagen mRNA isoforms in mouse. Dev. Dyn. Off. Publ. Am. Assoc. Anat..

[36] Yip C.Y., Chen J.H., Zhao R., Simmons C.A. (2009). Calcification by valve interstitial cells is regulated by the stiffness of the extracellular matrix. Arter. Thromb. Vasc. Biol..

[37] Baldinger A., Brehm B.R., Richter P., Bossert T., Gruen K., Hekmat K., Kosmehl H., Neri D., Figulla H.R., Berndt A. (2011). Comparative analysis of oncofetal fibronectin and tenascin-C expression in right atrial auricular and left ventricular human cardiac tissue from patients with coronary artery disease and aortic valve stenosis. Histochem. Cell Biol..

[38] Satta J., Melkko J., Pöllänen R., Tuukkanen J., Pääkkö P., Ohtonen P., Mennander A., Soini Y. (2002). Progression of human aortic valve stenosis is associated with tenascin-C expression. J. Am. Coll Cardiol..

[39] Kaden J.J., Dempfle C.E., Grobholz R., Fischer C.S., Vocke D.C., Kiliç R., Sarikoç A., Piñol R., Hagl S., Lang S. (2005). Inflammatory regulation of extracellular matrix remodeling in calcific aortic valve stenosis. Cardiovasc. Pathol..

[40] Mohler E.R., Gannon F., Reynolds C., Zimmerman R., Keane M.G., Kaplan F.S. (2001). Bone formation and inflammation in cardiac valves. Circulation.

[41] Liberman M., Bassi E., Martinatti M.K., Lario F.C., Wosniak J., Pomerantzeff P.M., Laurindo F.R. (2008). Oxidant generation predominates around calcifying foci and enhances progression of aortic valve calcification. Arter. Thromb. Vasc. Biol..

[42] Midwood K.S., Valenick L.V., Hsia H.C., Schwarzbauer J.E. (2004). Coregulation of fibronectin signaling and matrix contraction by tenascin-C and syndecan-4. Mol. Biol. Cell..

[43] Orend G., Huang W., Olayioye M.A., Hynes N.E., Chiquet-Ehrismann R. (2003). Tenascin-C blocks cell-cycle progression of anchorage-dependent fibroblasts on fibronectin through inhibition of syndecan-4. Oncogene.

[44] Yu X., Miyamoto S., Mekada E. (2000). Integrin α2 β1-dependent EGF receptor activation at cell-cell contact sites. J. Cell Sci..

[45] Gu X., Masters K.S. (2010). Regulation of valvular interstitial cell calcification by adhesive peptide sequences. J. Biomed. Mater. Res. A.

[46] Butcher J.T., Norris R.A., Hoffman S., Mjaatvedt C.H., Markwald R.R. (2007). Periostin promotes atrioventricular mesenchyme matrix invasion and remodeling mediated by integrin signaling through Rho/PI 3-kinase. Dev. Biol..

[47] Carrion K., Dyo J., Patel V., Sasik R., Mohamed S.A., Hardiman G., Nigam V. (2014). The long non-coding HOTAIR is modulated by cyclic stretch and WNT/β-CATENIN in human aortic valve cells and is a novel repressor of calcification genes. PLoS ONE.

[48] Zheng D., Wang B., Zhu X., Hu J., Sun J., Xuan J., Ge Z. (2019). LncRNA OIP5-AS1 inhibits osteoblast differentiation of valve interstitial cells via miR-137/TWIST11 axis. Biochem. Biophys. Res. Commun..

[49] Zhang L., Li G., Wang X., Zhang Y., Huang X., Wu H. (2021). lncRNA SNHG3 acts as oncogene in ovarian cancer through miR-139-5p and Notch1. Oncol. Lett..

[50] Dacheng W., Songhe L., Weidong J., Shutao Z., Jingjing L., Jiaming Z. (2020). LncRNA SNHG3 promotes the growth and metastasis of colorectal cancer by regulating miR-539/RUNX2 axis. Biomed. Pharm..

[51] Yang Y., Cai Y., Wu G., Chen X., Liu Y., Wang X., Yu J., Li C., Chen X., Jose P.A. (2015). Plasma long non-coding RNA, CoroMarker, a novel biomarker for diagnosis of coronary artery disease. Clin. Sci..

[52] Yan L., Zhang Y., Zhang W., Deng S.Q., Ge Z.R. (2020). LncRNA-NRF is a Potential Biomarker of Heart Failure After Acute Myocardial Infarction. J. Cardiovasc. Transl. Res..

