# Supplementary material for: Integrated Analysis of LncRNA-Mediated ceRNA Network in Calcific Aortic Valve Disease
Source: Cells. 2022 Jul 14;11(14):2204. doi: 10.3390/cells11142204 (PMC9315639; doi:10.3390/cells11142204)
Supplement: Supplementary file 1 [file cells-11-02204-s001.zip › cells-1740630-SI.pdf]

Supplementary files

Table S1. Clinical characteristics of patients for RT-qPCR analysis

	Non-mineralized valves	Calcific valves	P
Sample, n	20	30	
Age, years	55.3 ± 8.2	58.4 ± 3.83	0.08
Male, n (%)	12 (60.0)	18 (60.0)	1
BMI, Kg/m^2	22.6 ± 2.9	23.5 ± 2.7	0.27
Smoking, n (%)	10 (50.0)	13 (43.3)	0.64
Diabetes mellitus, n (%)	5 (25.0)	6 (20.0)	0.68
Hypertension, n (%)	8 (40.0)	9 (30.0)	0.47
Hyperlipidemia, n (%)	6 (30.0)	8 (26.7)	0.80
BAV, n (%)	0	3 (10)	0.16
LVEF, (%)	30.1 ± 7.4	64.5 ± 8.3	＜0.001
Statins, n(%)	6 (30)	8 (26.7)	0.80
β-Blockers, n (%)	12 (60)	16 (53.3)	0.64
ACEi/ARB, n(%)	11 (55.5)	18 (60)	0.73
Transvalvular pressure gradient (mmHg)	13.36 ± 3.82	73.24 ± 26.31	＜0.001
AVA, cm^2	3.69 ± 0.46	0.68 ± 0.21	＜0.001
AS, n (%)	0	30	＜0.001

Note: Values are means ± standard deviation or n (%).
Abbreviations: BMI, body mass index; LVEF, left ventricular ejection fraction; BAV, bicuspid aortic valve; AS, aortic stenosis, AVA, aortic valve area.

Table S2. The primer sequences for real-time polymerase chain reaction

**Primers for qPCR**	**Primer sequence (5′ to 3′)**
U6 forward	CGCTTCGGCAGCACATATAC
U6 reverse	TTCACGAATTTGCGTGTCATC
GAPDH forward	TGATGACATCAAGAAGGTGG
GAPDH reverse	TTGTCATACCAGGAAATGAGC
ZNF436-AS1 forward	GACTCCCACCCTCTTCAAGTT
ZNF436-AS1 reverse	AAATAACCGGGCACTGGGAA

Table S3. The differentiational expressed mRNAs downloaded from STRING was processed using MCODE for further gene cluster analysis. An analysis of gene clusters with top 5 scores was conducted and presented in a table below.

Cluster	Score (Density*#Nodes)	Nodes	Edges	Node IDs
1	36.873	72	1309	AGT, APLNR, ADCY4, APLN, CXCL9, OPRL1, PPBP, GPR37L1, SERPINH1, PLOD1, COL5A2, COL5A1, FPR2, ADORA3, P2RY14, CCL19, S1PR4, COL9A1, COL9A3, COL6A5, CXCL3, CXCL1, CXCR2, COL8A1, SAA1, COL3A1, CXCL11, CXCR1, COL2A1, S1PR5, CXCL12, COL12A1, COL1A2, COL1A1, COL4A4, COL4A3, COL11A1, P4HA3, COL4A1, COL22A1, COL4A2, CCR5, CCR4, HTR1B, COL6A6, CXCL5, AGTR2, NPY, CXCL13, ADRA2A, GRM7, COL18A1, CXCR6, GNB4, CXCL16, COL17A1, COL15A1, ADCY5, CCL13, COL13A1, CX3CR1, COL27A1, CCL21, CCR7, CXCR3, CXCL10, COL10A1, COL14A1, GPSM1, LPAR1, COL7A1, SSTR1
2	30.879	158	2424	CD163, CCL11, IL21R, CD209, HTR7, SCG2, TLR7, F5, CD38, TNFSF9, EVA1A, SLAMF1, EDNRA, CD69, GPR83, MMP13, BTLA, KALRN, GZMK, IDO1, ADM2, CALCR, GPHA2, TNC, GPR84, IGFBP3, GPR68, GPR29, CD3E, NTS, CD3D, IL1RN, IL11, PRF1, GZMB, LAG3, TNFRSF4, TNFSF4, TNFSF13B, HLA-DRA, TNFSF11, ADRA1A, CTLA4, CD80, CYR61, IGFBP7, APOA1, FASLG, ITGB4, FCGR2A, CD40LG, PDCD1, ADCYAP1R1, ADCYAP1, CCL8, ITGA8, STC2, CD247, ZAP70, IL7R, IL7, SPP1, OASL, CD5, NCAM1, CD48, STAT4, CD244, BCL6, EDNRB, FCGR1A, OXTR, CIITA, CD1C, JAK3, CD28, ITGA4, ITGB7, TIGIT, FCGR1B, CD226, IBSP, IRF6, IL1B, HTR2C, AGTR1, ITGAX, GZMA, DMP1, IL3RA, PNPLA2, CD8A, CR2, CD8B, VIPR1, MEPE, IRF7, IL2RA, PRDM1, IL2RB, ADORA2B, KLRB1, KISS1, GNRH1, HLA-DPA1, CD52, SERPINA1, LUM, HLA-DPB1, HLA-DQB2, GBP1, TBXA2R, UTS2, SELE, TNFRSF18, CD27, CD70, CXCL14, EOMES, ITGB3, FN1, LCK, OAS3, PRSS23, CD34, VCAM1, GLP2R, ITGAL,

				GPBAR1, GPR65, VEGFA, TLR10, TMEM132A, DRD1, PTGER4, PTGER2, CD7, IL6, CD2, ITGA10, ITGA11, CD83, ITK, C4A, CCL7, IGFBP1, IGFBP4, MMP9, HLA-DQA2, KLRC1, KLRD1, CD276, P2RY10, FAM20A, FAM20C, PCSK9, RXFP1, THY1
3	11.954	175	1040	ACACB, FASN, SLC2A3, IRS2, LIF, ACTG2, PTX3, LYPD1, KRT18, SLC27A6, PDK4, PRND, TNFAIP3, SOX2, SERPINF2, SH2D1A, LAMA4, ACAN, SERPINE2, NTF3, ADAMTS9, SREBF1, PRSS3, NTM, NEGR1, PGF, MYH9, ZBTB16, KLRF1, CLEC5A, CD6, ALCAM, GNLY, CIDEC, CIDEA, ANGPT2, GBP5, ADAMTS8, MYOCD, FGF1, NEB, MMP11, FCGR3A, SLC2A5, MYH14, SAMHD1, CDK6, CNN2, CCND1, PLAU, PLAUR, OSM, GPAM, SLAMF6, GZMH, SERPINE1, MLXIPL, CD74, SGCD, THSD7B, EMR1, TNFRSF11B, PPARGC1A, PTPRB, HBB, WNT5A, LPL, MMP1, CD14, CD93, DGAT2, VTN, GDNF, SEMA5B, RNF182, ITGA2, LAMB3, STBD1, METTL7A, LYPD3, CPM, SBSPON, DIO2, LAMA2, SLC2A4, FIGF, RAET1G, FLT4, CASP1, NLRP3, TRAT1, PNPLA3, BMP7, MMP25, SDC1, ACTC1, GFAP, CDKN2A, IL1R2, TNN, VASP, GLIS2, ACE, PLIN2, ADAMTS16, TREM1, FBXL22, NKG7, FBXL13, NT5E, ASB2, MYL3, ASB4, LIPE, SCD, OSCAR, CTGF, LMO7, DLL4, S100B, IFI6, LILRB1, CASQ2, PRKCQ, CD300A, JAG1, TIMP3, MME, RORC, TNFSF18, FGF22, PLIN1, EGR1, LYVE1, CD3G, SP7, FGF7, TNF, RUNX2, ADAM8, LY6H, ASPN, LYPD2, OLR1, M ICB, DPP4, HCST, THRSP, MGAM, HGF, CHAD, SPARC, LDB3, SOCS3, MYOZ2, FABP4, SKAP1, TRIM63, FBXO2, MS4A1, PCK1, CD79B, TNFRSF8, CD79A, ULBP1, CR1, ADAMTS12, TLL1, LAMC3, LAMA3, SPSB1, SEMA5A, ADAMTS15, THBS2, AR
4	6.862	175	597	PPP1R12B, GPT, LIFR, IL22RA1, TPM2, CACNG8, TNFAIP6, GRIN2D, TMEM26, F3, MFAP5, KIF26B, GUCY1A2, NR2F2, CRTAM, GRIA4, RBP4, AFAP1L1, GATA4, KIF1A, KIF5A, CALD1,

				KDELR3, SYN1, GDF15, TAGLN, MATK, TMOD1, PLAC8, CTSE, GUCY1A3, CNTFR, GUCY1B3, PADI2, GPD1, GPR174, HLA-DOA, PPARG, WIF1, TAGLN3, CNN1, ISG15, FZD8, IFIT1, ABCA1, FOXO3, FZD7, NLGN1, ID1, SH3PXD2A, RET, ROBO2, HEY1, SLIT3, NOTCH3, NKD1, CTSW, SFRP5, KIF21A, WISP1, NRCAM, KIF21B, IL12RB2, HTRA1, SEMA7A, CTHRC1, NPR1, PDE5A, PLN, FST, SHISA9, NME1, MAPT, RNASE2, RSAD2, NOG, ASPG, TPM1, MYLK, ENPP1, MYL9, CASP5, G6PD, MRAP, VDR, ITGBL1, GRIK4, BPI, CTSG, DLX5, NFASC, LY9, PKP2, CAMP, SOX10, RELN, JPH2, MYH7, SPTBN2, PHEX, EPHA1, MYOT, EFNB3, TGFB3, BAIAP2, ABCB1, FOSL1, MXRA5, JUNB, ADAMTS14, ITLN1, MCAM, TRDN, DLG2, SHANK1, GRID2, ADIPOQ, WNT9B, MYOZ1, RASGRF2, NLRC5, HIP1R, TCAP, CLEC11A, KRT14, CTSV, JAG2, CYFIP2, WASF3, IER2, ACTA2, RHOB, PDE4D, MYH11, EPHA7, CYP19A1, ADAM12, EPHB6, PLP1, EPHB1, UNC13A, JUP, PRLR, EGF, TNNT2, KIF5C, PDE6A, SCIN, FOSB, STMN2, AIM2, FOXO1, BTG2, SLAMF7, KIF19, CTSC, CFP, GLI3, CST7, PTCH1, CACNA2D3, MSTN, SEMA6B, RHOU, EGR3, EGR2, ERBB3, GAP43, TRAF3IP3, ELAVL4, TGFA, PLXNB3, PLD4, FRK, CATSPERB
5	4.8	6	12	AKR1C1, GPX8, AKR1C2, TXNRD1, NQO1, ALDH3A1

Table S4. The hub miRNAs and Its target genes.

miRNA	Genes targeted by miRNAs	Gene counts
has-let-7a-5p	COL9A1, THBS1, COL4A2	3
has-let-7e-5p	THBS1, ITGB3, COL4A1	3
has-miR-6766-3p	COL9A1, ITGA2	2
has-let-7d-5p	THBS1, COL1A1	2
has-miR-27b-3p	ITGA2, FN1	2
has-miR-106b-5p	COL4A1, COL4A3	2
has-miR-29a-3p	COL6A3, LAMA2	2
has-miR-148a-3p	ITGA11, LAMA4	2
has-miR-326	TNC, FRAS1	2
has-miR-25-3p	NPNT, ITGA8	2
has-miR-98-5p	COL4A1, COL4A2	2

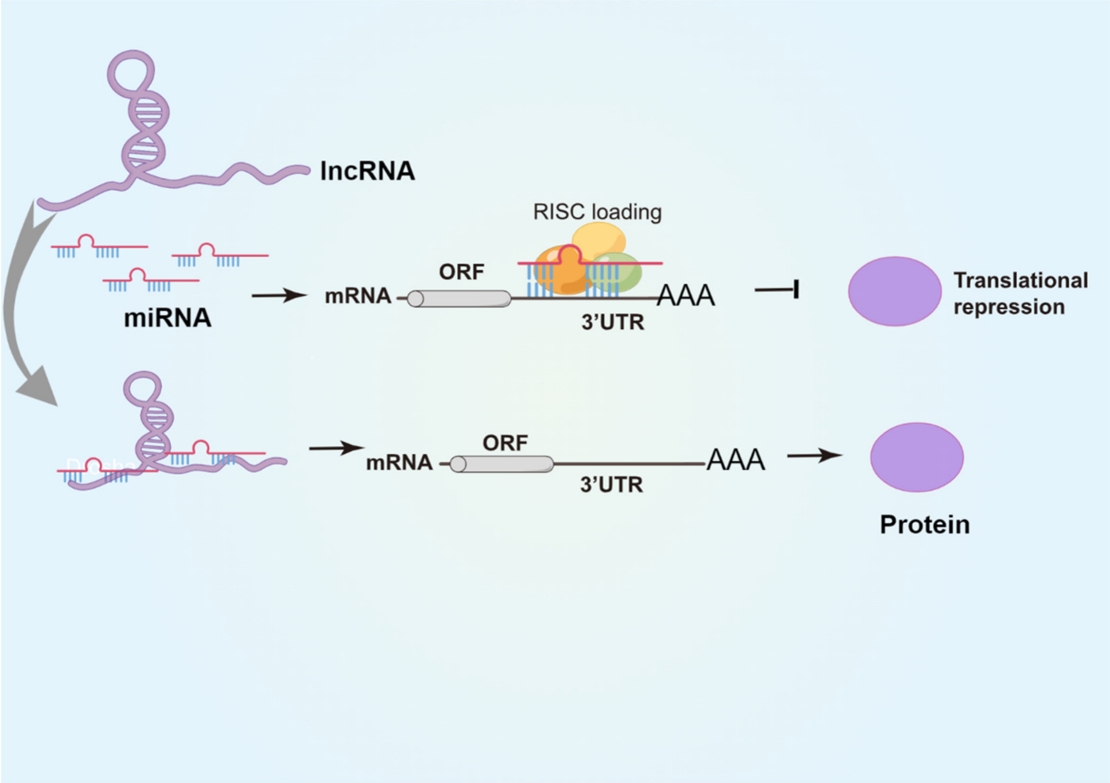

Supplementary Figure S1. The schematic diagram of lncRNA-mediated ceRNA regulatory network.
